# Calyceal Diverticulum: A Cystic Lesion Mimicking a Urinary Tract Infection

**DOI:** 10.7759/cureus.99468

**Published:** 2025-12-17

**Authors:** Miguel Lucas, Maria José Noruegas, Marta Machado

**Affiliations:** 1 Department of Pediatrics, Hospital Pediátrico, Unidade Local de Saúde de Coimbra, Coimbra, PRT; 2 Department of Medical Imaging, Unidade Local de Saúde de Coimbra, Coimbra, PRT; 3 Pediatric Nephrology Unit, Hospital Pediátrico, Unidade Local de Saúde de Coimbra, Coimbra, PRT

**Keywords:** calyceal diverticulum, diverticulum, kidney calices, urinary tract infection, urogenital abnormalities

## Abstract

Calyceal diverticulum (CD) is an uncommon renal anomaly in children and often mimics other cystic lesions, making diagnosis challenging. We report the case of a nine-year-old boy evaluated during follow-up after an episode of acute pyelonephritis. He remained asymptomatic, with normal examination findings and appropriate growth. Renal ultrasound revealed a non-vascularized cystic lesion with heterogeneous content in the upper pole of the right kidney. Subsequent magnetic resonance imaging demonstrated communication with the collecting system, confirming the diagnosis of CD. While typically asymptomatic, CD may initially present with complications such as urinary tract infection (UTI). Identification relies on imaging modalities capable of visualizing the diverticular communication with the calyceal system. Management in pediatric patients is generally conservative, with surgical intervention reserved for recurrent infection, abscess formation, persistent pain, gross hematuria, or progressive enlargement. This case highlights that UTI is a complication of calyceal diverticulum and could be its first clinical manifestation. It further emphasizes the importance of including CD in the differential diagnosis of atypical pediatric renal cystic lesions and reinforces the crucial role of advanced imaging in establishing a definitive diagnosis.

## Introduction

Calyceal diverticulum (CD) is a congenital or acquired outpouching of a renal calyx, lined by transitional epithelium and connected to the collecting system through a narrow channel [1-3]. Its anatomy often leads to urinary stasis, which promotes bacterial growth and stone formation [3]. It is rarely identified in children, with a reported prevalence ranging from 0.2% to 0.4% in both pediatric and adult populations [4]. Because it closely resembles other renal cystic lesions, its detection can be challenging [4-8]. In pediatric patients, ultrasound typically shows a well-defined cystic lesion within the renal parenchyma [4-8], often containing echogenic, mobile debris, its most distinctive feature [5]. When this feature is absent, differentiation from other cystic lesions generally requires contrast-enhanced imaging modalities [4-10], particularly renal magnetic resonance imaging (MRI), which is preferred in children [6,9,11]. Also, CD is usually solitary and may occur at any renal pole, although an upper-pole predominance has been described [10]. The identification of CD is important to rule out other cystic renal lesions, including complex cysts and, in rare cases, neoplastic processes [1,10,12].

## Case presentation

**Figure 1:**
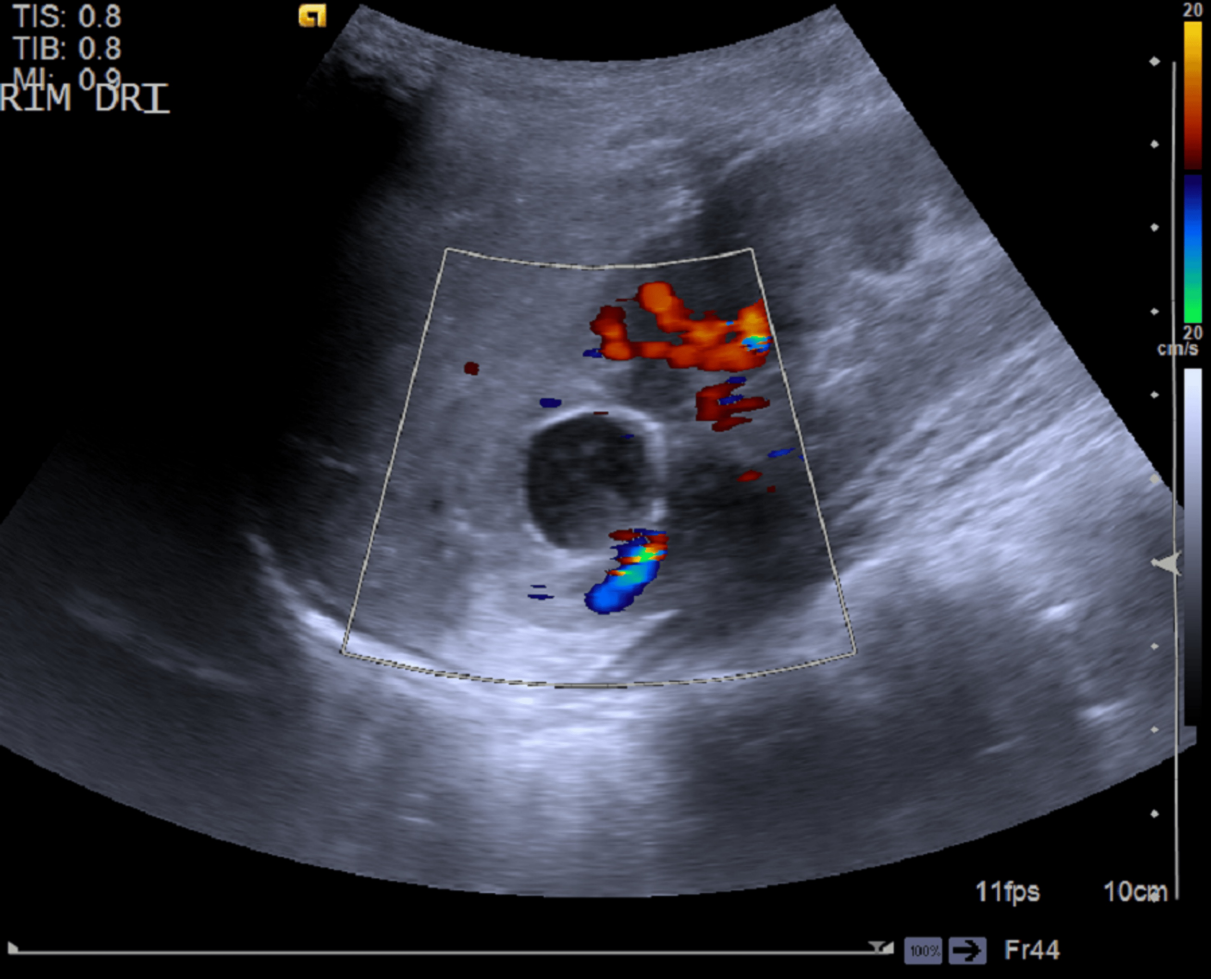
Ultrasound imaging showing a non-vascularized, thin-walled cystic formation with heterogeneous content in the upper third of the right kidney.

A nine-year-old boy was referred to the general pediatrics consultation for follow-up after an episode of acute pyelonephritis that occurred at eight years of age. He had a history of attention deficit hyperactivity disorder and was taking methylphenidate. The growth was regular, and the psychomotor development was adequate. His mother has recurrent renal colic. Clinically, he was asymptomatic, his blood pressure was 106/61 mmHg, he was below the 90th percentile for height and sex, and the physical examination was normal. A renal ultrasound revealed a non-vascularized, thin-walled cystic formation with heterogeneous content in the upper third of the right kidney (Figure 1).

**Figure 2:**
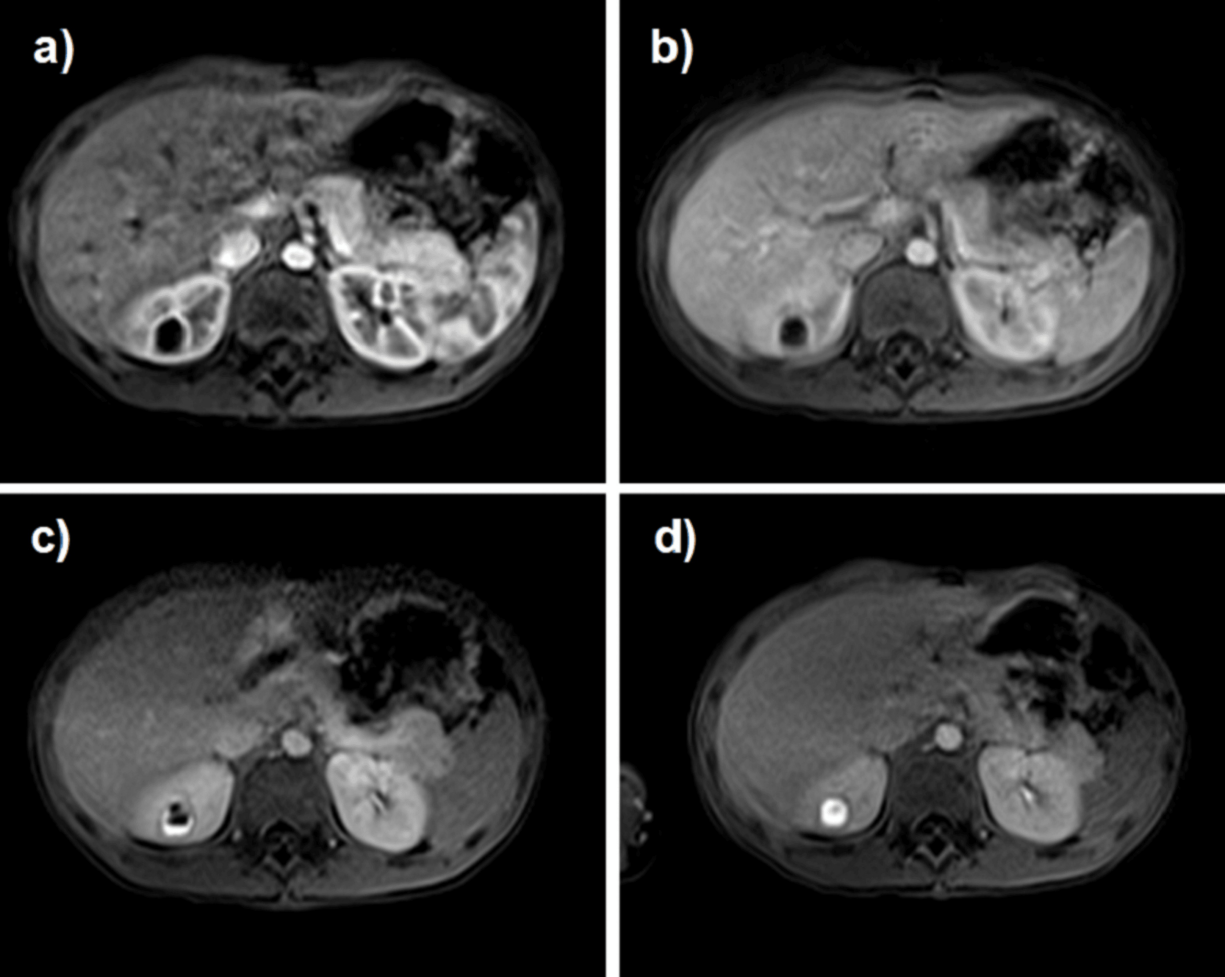
Magnetic resonance imaging (T1) showing a nodular lesion with peripheral enhancement in the arterial phase (a), progressive (b, c) and total enhancement in the excretory phase (d), confirming communication with the collecting system.

For better characterization, renal MRI was performed (Figure 2). The demonstration of contrast filling of the lesion in the delayed phase, confirming its communication with the collecting system, was consistent with a CD.

As no complications were identified, conservative management was chosen, and the patient has since been monitored through annual follow-ups without incident.

## Discussion

Typically asymptomatic, CD often remains undetected and, when identified, may mimic other renal cystic lesions, including simple and complex cysts and, in rare cases, neoplastic processes [10,12]. When symptomatic, it may present with urinary tract infection (UTI), abdominal or flank pain, hematuria, and, less commonly, hypertension or a palpable mass [13]. Its narrow communication with the calyx promotes urinary stasis, which in turn compromises effective drainage and the normal flushing of bacteria, thereby increasing susceptibility to recurrent UTIs [3]. In our case, UTI was the initial clinical manifestation, consistent with literature describing it as a possible complication and, in some instances, the first presenting feature [1,13].

Diagnosis relies on imaging that demonstrates the communication between the diverticular cavity and the collecting system, particularly through contrast studies and late-phase post-contrast images, which allow direct visualization of this connection and help differentiate CD from other renal cystic lesions [4-8]. In most cases, ultrasound alone is insufficient, as in our patient, because it may not clearly distinguish a calyceal diverticulum from a simple or complex cystic lesion (Figure 1). Consequently, renal MRI was performed, revealing a nodular lesion with peripheral arterial-phase enhancement and progressive, complete enhancement during the excretory phase, findings that confirmed the diagnosis of CD (Figure 2). Renal MRI plays a central role in diagnosing calyceal diverticulum in pediatric patients [6,9], providing high-resolution, multiplanar anatomical detail without ionizing radiation, an important advantage in children [11].

Conservative treatment in asymptomatic cases is generally preferred; this typically includes annual or biannual follow-up with clinical assessment (blood pressure, symptom review), urinalysis, and renal ultrasound to monitor for signs of recurrence, lithiasis, or size progression [10,13]. Surgery is rarely required, indicated in cases of recurrent UTI, abscess formation, increasing diverticular size, persistent pain, or gross hematuria [1,9]. Since no complications were identified, conservative management was chosen, and the patient has since been monitored through annual follow-ups without incident.

## Conclusions

CD is a rare pediatric renal anomaly that can mimic other cystic lesions, potentially delaying diagnosis. While typically asymptomatic, it may first present with a urinary tract infection, as illustrated in this case. Advanced imaging that demonstrates communication with the collecting system enables a definitive diagnosis, thereby guiding appropriate management and avoiding unnecessary interventions.
